# Prevalence, characteristics, and risk factors of non-alcoholic fatty liver disease in North East of Iran: a population-based study

**DOI:** 10.1186/s12876-024-03302-y

**Published:** 2024-06-26

**Authors:** Mina AkbariRad, Masoud Pezeshki Rad, Hadi Nobakht, AmirAli Moodi Ghalibaf, Abdollah Firoozi, Ashkan Torshizian, Amir Reza Bina, Ali Beheshti Namdar, Masoumeh Sadeghi

**Affiliations:** 1 Department of Internal Medicine, Faculty of Medicine, Mashhad University of Medical Sciences, Mashhad, Iran; 2 Department of Radiology, Faculty of Medicine, Mashhad University of Medical Sciences, Mashhad, Iran; 3 Faculty of Medicine, Mashhad University of Medical Sciences, Mashhad, Iran; 4 Student Research Committee, Birjand University of Medical Sciences, Birjand, Iran; 5 Mashhad University of Medical Sciences, Mashhad, Iran; 6 Metabolic Syndrome Research Center, Mashhad University of Medical Sciences, Mashhad, Iran; 7 Department of Gastroenterology, Faculty of Medicine, Mashhad University of Medical Sciences, Mashhad, Iran; 8 Department of Epidemiology, School of Health, Mashhad University of Medical Sciences, Mashhad, Iran

**Keywords:** NAFLD, Non-alcoholic fatty liver disease, Liver disease, Risk factors, Epidemiology

## Abstract

**Background:**

Nonalcoholic fatty liver disease (NAFLD) is a common dietary disorder caused by fatty changes in the liver parenchyma and hepatocytes without alcohol consumption. The present study aimed to investigate the prevalence, characteristics, and risk factors of NAFLD in the Mashhad Persian Cohort Study population.

**Method:**

The present population-based cross-sectional study included all PERSIAN Organizational Cohort study in Mashhad University of Medical Sciences (POCM), Mashhad, Iran by census sampling method. Eligible participants were divided into two groups due to their NAFLD condition (NAFLD positive or NAFLD negative). All enrolled participants were evaluated based on their clinical aspects, anthropometric measures, laboratory tests, and ultrasound features. Statistical analysis was conducted using SPSS software version 16 (SPSS Inc., Chicago, USA –version 16). A P-value less than 0.05 was considered as the significance level.

**Results:**

A total of 1198 individuals were included in the study, of which 638 (53.3%) were male and the rest were female. The mean age of the participants was 46.89 ± 8.98 years. A total of 246 patients (20.53%) were NAFLD positive, of which 122 (49.59%) were in grade 1, 112 (45.52%) were in grade 2, and 12 (4.87%) were in grade 3. The prevalence of fatty liver was significantly higher in males than in females (*p* < 0.001). There were significant differences between NAFLD positive and NAFLD negative participants in terms of having a history of hypertension (*P* = 0.044), body mass index (*P* < 0.001), body fat percentage (*P* = 0.001), waist circumference (*P* < 0.001), liver craniocaudal length (*P* = 0.012), fasting blood sugar (FBS) (*P* = 0.047), aspartate aminotransferase (AST) (*P* = 0.007), and alanine aminotransferase (ALT) (*P* = 0.001). Further analysis revealed a strong significant association between BMI, previous history of hypertension, higher levels of serum ALT, and NAFLD (*P* < 0.05).

**Conclusion:**

It can be concluded that ultrasound findings accompanied by laboratory AST and ALT level enzymes could be a cost-benefit approach for NAFLD early diagnosis. The craniocaudal size of the liver could be a beneficent marker for estimating the severity of the disease; however, more studies are recommended to evaluate this variable for future practice against the issue.

## Introduction

Near 40 years ago, the term “nonalcoholic steatohepatitis (NASH)” was proposed by Ludwig et al., that present mimic alcoholic hepatitis histologically without a significant alcohol consumption [1]. Further studies by Schaffner et al (1986) presented a similar histopathological condition caused by no significant alcohol consumption as “nonalcoholic fatty liver disease (NAFLD)” [2]. Despite alcohol consumption being known as the main cause of fatty-liver disease incidence, NAFLD is commonly associated with metabolic disorders including glucose tolerance impairment, central obesity, hypertension, hypertriglyceridemia, and low serum levels of high-density lipoprotein (HDL) [3–5]. In the past decades, NAFLD had been diagnosed by ruling out other causes of fatty liver, especially excessive alcohol intake; however, further investigations indicated that NAFLD is not only associated with liver-related complications but also can potentially be related to metabolic disorders, renal and cardiovascular complications [6]. Beyond the NAFLD’s complications, its incidence and prevalence are too high all over the world; as the last reports stated the NAFLD as one of the major worldwide health problems, not only affect more than 25% (approximately 1 billion individuals) of the adult general population but also its prevalence increase every day [7]. Generally, the major influence areas of the NAFLD are the Middle East and South America, while it has the least prevalence in Africa [8]. Studies have been determined that more than 100 million people in the United States and near half of the European citizens are suffer from NAFLD and its complications [9, 10]. Despite several studies have been reported various ranges of NAFLD prevalence in the Iranian general population from 7 to 45%, almost many studies unanimous that this metabolic disorder prevalence is increased approximately 40% in the past two decades [11, 12]. This increase with a steep slope can be due to the people’s lifestyle changes, as the NAFLD major knew risk factors are obesity, diabetes mellitus, dyslipidemia, and low HDL levels [13]; Moreover, some other conditions and disorders such as viral hepatitis, hypothyroidism, polycystic ovary syndrome, and smoking are under investigations as the possible risk factors of the NAFLD [13–15]. A focus on the Iranian adult general population indicated a wide range of the NAFLD prevalence in various regions of the country; however, there is a lack of information about many areas of the country due to a lack of accurate reports [16]. NAFLD not only affects people’s health but also imposes high amounts of costs on the people and governments, which is estimated approximately more than 100 billion dollars for a year [17]. Obviously, epidemiological studies are one of the basic ways to determine the status of the disease burden in a specific region which can potentially utilize for policy-making. Furthermore, due to the lack of novel accurate information about the NAFLD prevalence status in the NorthEast of Iran, and the high rate of NAFLD prevalence increase in the world, we have been designed the present study to investigate the prevalence, characteristics, and risk factors of the NAFLD in the second large city of Iran.

## Methods

### Study design

The present population-based, cross-sectional study was conducted within the framework of the PERSIAN Organizational Cohort study in Mashhad University of Medical Sciences (POCM), Mashhad, Iran in 2021 [18, 19]. All participants of the POCM in 2021 were assessed by the census sampling method. Informed consent was obtained from all participants of the study. All procedures of this study were in line with the ethical principles of Helsinki and were approved by the ethics committee of Mashhad University of Medical Sciences, Mashhad, Iran (IR.MUM.MEDICA.REC.1397.750).

### Study population and procedure

All PCOM adult population between 20 and 75 years old, who have been completed the written informed consent, were included in the study. Furthermore, all of them were personnel of the university. On the other hand, the study exclusion criteria were considered as patients with liver failure, hepatitis (viral, autoimmune, metabolic, pharmacological), a history of alcohol consumption (more than 20 gr daily for women and 30 gr daily for men), serum alanine transaminase (ALT) and Aspartate transaminase (AST) level 5 times higher than normal values, being pregnant, receiving medications that can cause hepatic steatosis including antiarrhythmic medications (amiodarone), antimetabolite medications for cancer treatment (methotrexate), hormonal antineoplastic medications (tamoxifen), corticosteroids, anticonvulsant medications, and antiretroviral medications. After the participants fill out the written informed consent, their data were recorded through a checklist which has been included the demographic chrematistics (age, gender, weight, height, and waist circumference (WC)), body mass index (BMI) [18, 19], previous medical history, and underlying disease (such as diabetes mellitus, hypertension, etc.), smoking and opium addiction (Cigarettes and/or hookah), alcohol consumption, systolic blood pressure (SBP), diastolic blood pressure (DBP), fasting blood sugar (FBS), blood serum levels of triglycerides, total cholesterol, AST, ALT, alkaline phosphatase (ALP), and Gamma-glutamyltransferase (GGT). All biochemical tests including blood serum levels of the FBS, AST, ALT, ALP, GGT, triglycerides, and total cholesterol were measured by Biotecnica BT 1500^®^ - Chemistry Analyzer. Finally, all subjects underwent abdominal sonography; especially hepatobiliary (liver and biliary tract) ultrasonography was performed for all included patients by the approved expert sonographer through utilizing Philips Affiniti 50 Ultrasound Machine^®^. Fatty liver is diagnosed based on the following ultrasound parameters: parenchymal brightness, liver-to-kidney contrast, deep beam attenuation, bright vessel walls, and gallbladder wall definition. In fact, fatty liver was diagnosed with an increase in hepatic echogenicity using renal echogenicity as a reference, the presence of enhancement, and a lack of differentiation of periportal and bile duct walls reinforcement because of great hyperechogenicity of the parenchyma [18, 20]. Hepatic steatosis is graded as follows: Absent (score 0) when the echotexture of the liver is normal; mild (score 1), when there is a slight and diffuse increase of liver echogenicity with normal visualization of the diaphragm and of the portal vein wall; moderate (score 2), in case of a moderate increase of liver echogenicity with the slightly impaired appearance of the portal vein wall and the diaphragm; severe (score 3), in case of the marked increase of liver echogenicity with poor or no visualization of the portal vein wall, diaphragm, and posterior part of the right liver lobe [21].

### Statistical analysis

Statistical analysis was conducted using SPSS software version 16 (SPSS Inc., Chicago, USA –version 16). Descriptive statistics for the demographic characteristics were presented by the frequency (percentage) for qualitative variables, and measures of central tendency (mean and standard deviation) for quantitative variables. Age standardized prevalence rate was calculated using direct standardization method and the 2021 Iranian population age structure provided by the United Nations (available at https://population.un.org/wpp/Download/Standard/Population) as the reference, while the confidence interval was calculated using the Keyfitz method [22]. The Chi-square test was used to investigate the relationship between qualitative variables. The t-test or Mann–Whitney U test was applied for the normal distributed quantitative variables, while Kruskal–Wallis test (one-way ANOVA) or Wilcoxon test was used for non-parametric quantitative variables. Moreover, Univariate and multivariable ordinal logistic regression analyses were performed to comprehensively assess the association of demographic, anthropometric, and laboratory data, along with physical examination findings, with different grades of NAFLD among the entire study population including healthy subjects (grade 0), and subjects with grade I, II, and III NAFLD. Adjusted odds ratios with 95%Confidence Interval (CI) were presented for potential determinant factors of severity levels of NAFLD. For ordinal regression analysis, BMI was employed in the analysis as a categorical variable. BMI categories included normal weight (BMI 18.5 to 24.9 kg/m²), overweight (BMI 25 to 29.9 kg/m²), and obese (BMI 30 kg/m² and above). Variable selection for the multivariate model was based on clinical relevance or a *P* < 0.25 in univariate analysis. At last, multicollinearity among variables was assessed using various linear regression models. In cases where two variables were identified as multicollinear (e.g., WC and BMI), only the one deemed to have higher clinical significance was included in the final regression model. P-values less than 0.05 were considered statistically significant for all tests. All Statistical analysis was conducted using Stata11.0 (StataCorp, Texas, US).

## Results

### Participants characteristics

Of a total of 1198 (from 5287 individuals in the POCM study (22.65%)) participants were enrolled in this cross-sectional POCM-based study, and the majority of participants (53.8%) were males. The crude prevalence rate of NAFLD was 205 per 1000 people. After proper age adjustment, this rate dropped to 181 (95% CI: 160–202) per 1000 people. The mean age of NAFLD positive and NAFLD negative participants were 46.41 ± 7.37 and 47.01 ± 9.35, respectively, The two groups did not differ significantly in regards to their age (*P* = 0.282); however, due to the age groups, NAFLD positive cases were mostly middle-aged and NAFLD negatives belonged to the young and elders (*P* < 0.001). Due to the study population data analysis, the history of underlying chronic conditions were as follows: diabetes mellitus (132; 11%), hypertension (224; 18.7%), and metabolic syndrome (262; 21.9%) (Table 1).

**Table 1:** Demographic and objective assessments’ results of all study participants (*N* = 1198)

Variables		Study Population	NAFLD^b^+ (*N* = 246)	NAFLD^b^– (*N* = 952)		*P*-value^c^
*Demographic characteristics*						
**Age (years)**		46.89 ± 8.98	46.41 ± 7.37		47.01 ± 9.35	0.282^+^
**Age categories (years)**	**30–39**	333 (27.8)	53 (15.9)		280 (84.1)	**< 0.001** ^**#**^
	**40–49**	429 (35.8)	110 (25.6)		319 (74.4)	
	**50–59**	295 (24.6)	70 (23.7)		225 (76.3)	
	**60–69**	132 (11.0)	13 (9.8)		119 (90.2)	
	**70–79**	9 (7.6)	0 (0.0)		9 (100)	
Gender*	**Male**	638 (53.3)	154 (24.1)		484 (75.8)	**0.001** ^**#**^
	**Female**	560 (46.7)	92 (16.4)		468 (83.5)	
Hx* ^**a**^	**Diabetes Mellitus**	132 (11.0)	22 (16.7)		110 (83.3)	0.244^#^
	**Hypertension**	224 (18.7)	37 (16.5)		187 (83.5)	0.099^#^
	**Cigarette smoking**	124 (10.8)	22 (17.7)		102 (82.3)	0.591^#^
	**Hookah smoking**	92 (8.0)	19 (20.7)		73 (79.3)	0.375^#^
	**Metabolic syndrome**	262 (21.9)	52 (19.9)		210 (80.1)	0.796^#^
*Physical examination & imaging findings*						
**Systolic blood pressure (mmHg)**		127.55 ± 16.6	128.87 ± 15.64		127.22 ± 16.86	0.174^+^
**Diastolic blood pressure (mmHg)**		82.69 ± 12.57	84.32 ± 11.38		82.28 ± 12.82	**0.017** ^**+**^
**Body mass index (kg/m** ^**2**^ **)**		28.95 ± 4.10	29.4 ± 3.9		28.8 ± 4.1	**0.029** ^**+**^
**Body fat percentage**		28.6 ± 8.5	29.1 ± 8.3		28.5 ± 8.5	0.293^+^
**Waist circumference (cm)**		98 ± 10	100.4 ± 10.1		97.9 ± 10.7	**0.001** ^**+**^
**Liver craniocaudal length (mm)**		109.53 ± 15.53	110.99 ± 16.97		107.86 ± 13.58	0.077^+^
*Laboratory markers*						
**Fasting blood sugar (** $\text{m}\text{g}/\text{d}\text{L}$ **)**		101.98 ± 27.37	101.44 ± 24.47		102.12 ± 28.06	0.711^+^
**Triglyceride (** $\text{m}\text{g}/\text{d}\text{L}$ **)**		146.35 ± 82.45	155.82 ± 89.87		143.95 ± 80.36	**0.047** ^**+**^
**Cholesterol** $(\text{m}\text{g}/\text{d}\text{L}$ **)**		183.19 ± 37.68	184.92 ± 34.43		182.76 ± 38.47	0.791^+^
**Aspartate aminotransferase (** $\text{u}\text{n}\text{i}\text{t}\text{s}/\text{L}$ **)**		24.51 ± 10.35	25.91 ± 11.19		24.15 ± 10.11	**0.019** ^**+**^
**Alanine aminotransferase (** $\text{u}\text{n}\text{i}\text{t}\text{s}/\text{L}$ **)**		31.49 ± 16.76	34.67 ± 17.87		30.69 ± 16.38	**0.001** ^**+**^
**Alkaline phosphatase (** $\text{u}\text{n}\text{i}\text{t}\text{s}/\text{L}$ **)**		184.75 ± 74.71	189.88 ± 76.92		183.45 ± 74.13	0.235^+^
**Gamma-glutamyl transferase (** $\text{u}\text{n}\text{i}\text{t}\text{s}/\text{L}$ **)**		33.81 ± 28.36	35.23 ± 29.77		33.45 ± 28.00	0.388^+^

*Qualitative variables were presented as “Number (Percentage)” and the others have shown in the format of “Mean ± SD (Standard Deviation)” as they were quantitative; ^#^Fisher exact test has been performed; ^+^Independent T-test analysis was done; ^a^History; ^b^Non-alcoholic fatty liver disease; ^c^This value has been calculated through mentioned analyses just between cases (NAFLD positives) and controls (NAFLD negatives)

Among 1198 participants, 246 (20.53%) were NAFLD-positive cases who were diagnosed during these assessments. DBP, BMI, and WC were also substantially higher in NAFLD cases compared to non-NAFLD participants (*P* < 0.05); whereas, SBP, body fat percentage (BFP), and liver craniocaudal length (LCL) had shown no association in the analysis (*P* > 0.05) (Table 1).

In the set of laboratory markers, triglyceride, AST, and ALT levels were meaningfully more in NAFLD-positives than NAFLD-negatives (*P* < 0.05) (Table 1).

### NAFLD-positive participants’ analysis

Based on data acquired from ultrasound assessments, among 246 NAFLD-positive cases, 122 cases (49.59%) were diagnosed as grade I, whereas 112 (45.53%) and 12 cases (4.8%) were diagnosed as second and third-grade NAFLD, respectively. Using subgroup analyses to identify factors associated with higher grades of NAFLD revealed that BMI, BFP, WC, LCL, FBS, AST, and ALT were significantly higher in higher grades compared to the lowers (*P* < 0.05). Despite SBP, DBP, triglyceride, and cholesterol levels not being different between the subgroups of the NAFLD-positive cases, pre-existing hypertension was significantly different between them (*P* > 0.05) (Table 2).

**Table 2:** Demographic and objective assessments’ results* of the POCM^$^ cases based on 3 grades of NAFLD^b^ (*N* = 1198)

Variables		Grade 1 (*N* = 122)	Grade 2 (*N* = 112)	Grade 3 (*N* = 12)	*P*-value^c^
*Demographic characteristics*					
**Age (years)**		46 ± 7	46 ± 6	46 ± 8	0.773^+^
**Age categories (years)**	**30–39**	31 (25.5)	18 (16.1)	4 (33.3)	0.414^#^
	**40–49**	48 (39.3)	58 (51.8)	4 (33.3)	
	**50–59**	36 (29.5)	31 (27.7)	3 (25.0)	
	**60–69**	7 (5.7)	5 (4.5)	1 (8.4)	
**Gender**	**Male**	70 (57.4)	74 (66.1)	10 (83.3)	0.122^#^
	**Female**	52 (42.6)	38 (33.9)	2 (16.7)	
**Hx** ^**a**^	**Diabetes Mellitus**	10 (8.2)	11 (9.8)	1 (8.3)	0.907^#^
	**Hypertension**	24 (19.7)	10 (8.9)	3 (25.0)	**0.044** ^**#**^
	**Cigarette smoking**	10 (8.5)	10 (9.9)	2 (28.6)	0.223^#^
	**Hookah smoking**	7 (6.0)	12 (11.9)	0 (0)	0.212^#^
	**Metabolic syndrome**	20 (16.4)	28 (25.0)	4 (33.3)	
*Physical examination, Anthropometric & imaging findings*					
**Systolic blood pressure (mmHg)**		128.61 ± 17.26	129.18 ± 14.19	128.64 ± 11.55	0.963^+^
**Diastolic blood pressure (mmHg)**		83.63 ± 12.62	84.71 ± 10.07	87.82 ± 9.23	0.453^+^
**Body mass index (kg/m** ^**2**^ **)**		28.61 ± 3.52	30.05 ± 4.23	32.6 ± 2.1	**< 0.001** ^**+**^
**Body fat percentage**		27.34 ± 7.84	30.29 ± 8.56	35.45 ± 6.08	**0.001** ^**+**^
**Waist circumference (cm)**		97.6 ± 9.9	102.4 ± 9.3	109.7 ± 8.6	**< 0.001** ^**+**^
**Liver craniocaudal length (mm)**		104.69 ± 12.93	110.94 ± 13.41	119.00 ± 21.21	**0.012** ^**+**^
*Laboratory markers*					
**Fasting blood sugar (** $\text{m}\text{g}/\text{d}\text{L}$ **)**		97.63 ± 13.67	105.31 ± 32.46	105.18 ± 20.01	**0.047** ^**+**^
**Triglyceride (** $\text{m}\text{g}/\text{d}\text{L}$ **)**		145.23 ± 98.51	168.03 ± 80.59	151.55 ± 62.46	0.158^+^
**Cholesterol** $(\text{m}\text{g}/\text{d}\text{L}$ **)**		181.99 ± 33.48	188.28 ± 34.58	183.82 ± 43.12	0.387^+^
**Aspartate aminotransferase (** $\text{u}\text{n}\text{i}\text{t}\text{s}/\text{L}$ **)**		23.89 ± 8.57	27.49 ± 12.12	32.36 ± 20.27	**0.007** ^**+**^
**Alanine aminotransferase (** $\text{u}\text{n}\text{i}\text{t}\text{s}/\text{L}$ **)**		30.41 ± 16.42	38.50 ± 17.81	43.45 ± 23.23	**0.001** ^**+**^
**Alkaline phosphatase (** $\text{u}\text{n}\text{i}\text{t}\text{s}/\text{L}$ **)**		187.53 ± 94.30	191.24 ± 54.33	202.18 ± 55.99	0.809^+^
**Gamma-glutamyl transferase (** $\text{u}\text{n}\text{i}\text{t}\text{s}/\text{L}$ **)**		34.71 ± 37.44	35.43 ± 19.85	38.91 ± 13.25	0.901^+^

^$^PERSIAN Organizational Cohort study in Mashhad University of Medical Sciences *Qualitative variables were presented as “Number (Percentage)” and the others have shown in the format of “Mean ± SD (Standard Deviation)” as they were quantitative; ^#^Chi-square test has been performed; ^+^ANOVA analysis was done; ^a^History; ^b^Non-alcoholic fatty liver disease; ^c^This value has been calculated through mentioned analyses just between cases (NAFLD positives) and controls (NAFLD negatives)

### Association of clinically important variables and NAFLD

Table 3 illustrates results from the ordinal logistic regression analysis assessing the association between NAFLD and clinically important variables. This analysis was conducted on the entire study population including patients with and without NAFLD. LCL, TG, ALP, HDL, and smoking status were not statistically significant for any category of NAFLD in univariate analysis and were therefore not employed in the multivariate model (*P* > 0.25 for all variables; data not shown in the table).

In the final adjusted regression model, female sex was found to be inversely associated with NAFLD, while overweight and obese patients were more likely to be categorized as having higher grades of NAFLD. Additionally, higher levels of ALT and a positive history of hypertension exhibited a statistically significant association with NAFLD (*p* < 0.05) (Table 3).

## Discussion

The present cross-sectional study has been conducted on information gathered from participants of the POCM found that 246 NAFLD cases were diagnosed during these assessments and they accounted for 20.53% of the total study population (*n* = 1198). Among demographic measures, gender and age categories were found to have significant associations with NAFLD existence. We observed that patients with a previous history of hypertension, obesity, and higher levels of serum ALT were more likely to have higher grades of NAFLD while female sex was inversely associated with NAFLD grades. About 6.3 to 33% of people all around the world are estimated to suffer from NAFLD with even higher prevalence in people with existing comorbidities [23].

In our study, 20.83% of the total participants were diagnosed with NAFLD through ultrasound findings of the hepatobiliary tract. The prevalence rate was dropped to 18.1% (95% CI: 16-20.2%) after age standardization. In 2016, Moghaddasifar et al. conducted a comprehensive systematic review and meta-analysis, synthesizing data from 11 studies that investigated the prevalence of NAFLD among Iranian adults. The meta-analysis estimated that approximately 33.7% (95% CI: 20.7-46.7%) of Iranian adults have NAFLD [23]. Our findings suggest a much lower prevalence. The present study study targets a more informative population with better socioeconomic status compared to the general Iranian population. NAFLD is believed to be more prevalent in populations with a lower socioeconomic status [24]. Moreover, in line with our current findings, large meta-analysis studies have demonstrated a higher prevalence of NAFLD in male populations compared to females [25]. Differences between the previous findings of Moghaddasifar et al. and the current study may therefore be rooted in the fact that their estimates were not based on either socioeconomic status or gender differences.

Up to our knowledge, this was the first study evaluating the NAFLD situation in academic employees. All contributors were adults and their mean age was 46.89 ± 8.98. In univariate analysis, gender, DBP, BMI, WC, LCL, triglyceride, AST, and ALT were significant factors in the analysis between the two main groups of the study. Being male and having higher levels of all mentioned quantitative markers were associated with NAFLD existence.

Smoking was one of the controversial factors among the previous studies that currently is not supported by the results, and it seems that a cross-sectional report of smoking history is not sufficient for making a judgment about its risks as people could change their habits throughout life [26]; and longitudinal cohort investigations are recommended to clearly find out the cause-and-effect relationship between the consumption of different kinds of addictive substances in different routes and amounts, and NAFLD incidence.

BMI is also linked strongly to NAFLD since 80% of patients with NAFLD have obesity and just 16% of them are in the normal BMI range without underlying metabolic risks [27, 28]. Another study on obese individuals revealed that insulin resistance and WC are also independently associated with fatty liver. WC which represents the amount of visceral adipose was stronger than BMI in relationship with NAFLD and metabolic syndrome [29]. A large prospective cohort study including over 21 thousand people from northern China has shown that both SBP and BMI are responsible for the development of NAFLD. Their mediation analysis revealed SBP is responsible for a significant part of the relationship between BMI and NAFLD [30]. LCL was also another significant factor in our study that was substantially related to the presence of NAFLD and higher degrees of fatty liver. Few studies have considered this variable in their investigation. One of them was Khanal et al. correlational cross-sectional research that found out the liver craniocaudal size (or LCL) was significantly higher in severe patients compared to mild cases [31].

When came to subgroup analysis between different three grades of NAFLD, our study showed that all the following factors are associated positively with NAFLD severity: obesity, previous history of hypertension, higher levels of serum ALT. A Korean study at the Health Promotion Center of Jeju National University Hospital from 2009 to 2017 on more than 7800 subjects reported that people with higher degrees of NAFLD (2 or 3) were mostly male and had metabolic syndrome compared to mild NAFLD patients and healthy individuals. BMI, FBS, cholesterol, triglyceride, AST, ALT, and ALP were also elevated in patients with higher degrees [32]. Another analytical cross-sectional study based on PERSIAN Guilan Cohort Study (PGCS) resulted that just ALT, AST, and GGT levels were higher significantly in severe cases of NAFLD [33]. Some other studies have also reported different results mostly from liver function tests (LFT) and it is necessary to research more on associated factors and to pool all results in meta-analyses to reach a clearer understanding of severe NAFLD cases [23, 34, 35]. In line with our findings, Song et al. revealed that higher levels of blood pressure are largely driven by the coexistence of moderate/severe NAFLD [36].

The strength of the present study is that it included a large population. It’s limitation was method of diagnosis at first that ultrasonography is highly sensitive (89%) and specify (93%) in detecting liver steatosis [37], but false-negatives are possibly present and has impacted the results as it was used in previous researches and we found controversies in their results. Gold standard approach for diagnosis of NAFLD is liver biopsy analysis which is not available in routine check-up centers [38, 39] and this method is invasive and has the risks of following complications [40]. Moreover, selection bias cannot be ruled out our study population were staff of the university and this sample could not represent the health status of all Iranian population, except employees of governmental organizations, and further information could better understand the current situation of this disease. In addition, we had not gathered the participants data regarding their diet while accumulating evidence has concluded that consumption of diets with high calory content, increase the incidence of NAFLD [41].

## Conclusion

Our study finally concluded that NAFLD is a prevalent disease in the POCM population. Fatty liver is highly associated with anthropometric features and individuals with high values of BMI and WC should be screened necessarily to prevent further liver damage and difficult-to-treat complications. Ultrasound findings accompanied by laboratory AST and ALT level enzymes could be a cost-benefit approach for NAFLD early diagnosis. The craniocaudal size of the liver could be a beneficent marker for estimating the severity of the disease; however, more studies are recommended to evaluate this variable for future practice against the issue.

**Table 3:** Association of important demographic, anthropometric, and laboratory characteristics with NAFLD (Univariate and multivariable ordinal logistic regression)

Variables	Univariate			Adjusted	
	OR (95% CI)	*P*-Value	OR (95% CI)		*P*-Value
**Age**, year	0.99 (0.97-1.00)	0.38	1.00 (0.98–1.02)		0.78
**Sex**, male as references					
Male	1	0.001	1		*0.002*
Female	0.66 (0.45–0.80)		0.60 (0.43–0.83)		
**BMI**, kg/m2					
< 18.5 (underweight)	1		1		
18.5–24.9 (normal weight)	1.68 (1.03–2.74)	0.04	1.65 (0.82–2.71)		*0.06*
25-29.9 (overweight)	2.0 (1.20–3.44)	0.008	2.21 (1.29–3.78)		*0.004*
≥ 30 (obese)	1.84 (0.94–3.62)	0.07	2.07 (1.01–4.19)		*0.05*
**Alchol consumption**					
No	1		1		
Yes	0.25 (0.32–1.86)	0.17	0.11 (0.02–1.44)		0.11
**Hypertension**					
No	1		1		
Yes	0.70 (0.48–1.03)	0.07	0.61 (0.38–0.97)		*0.03*
**Diabetes Mellitus**					
No	1		1		
Yes	0.76 (0.47–1.23)	0.25	0.99 (0.43–1.31)		0.32
**AST**, units⁄lit	1.01 (1.007–1.02)	< 0.001	1.01 (0.97–1.03)		0.41
**ALT**,units/lit	1.02 (1.005–1.03)	0.006	1.02 (1.01–1.03)		*0.04*

BMI; Body mass index, ALT; Aspartate aminotransferase, AST; Alanine aminotransferase

## Data Availability

The data that support the findings of this study are available on request from the corresponding author. The data are not publicly available due to privacy or ethical restrictions.

## Abbreviations

NAFLD: Non-alcoholic fatty liver disease
POCM: PERSIAN Organizational Cohort study in Mashhad University of Medical Sciences
FBS: Fasting blood sugar
AST: Aspartate aminotransferase
ALT: Alanine aminotransferase
ALP: Alkaline phosphatase
NASH: Nonalcoholic steatohepatitis
HDL: High-density lipoprotein
WC: Waist circumference
BMI: Body mass index
SBP: Systolic blood pressure
DBP: Diastolic blood pressure
GGT: Gamma-glutamyltransferase
BFP: Body fat percentage
LCL: Liver craniocaudal length
WHtR: Waist-to-height ratio
HC: Hip circumference
PGCS: PERSIAN Guilan Cohort Study
LFT: Liver function tests
